# Balancing Conductivity and Morphology in Aniline-Tuned Biopolymer–Starch Composites

**DOI:** 10.3390/polym17040497

**Published:** 2025-02-14

**Authors:** Mohammed E. Ali Mohsin, Suleiman Mousa

**Affiliations:** Department of Chemical Engineering, College of Engineering, King Faisal University, Al Ahsa 31982, Saudi Arabia

**Keywords:** biopolymer, polyaniline (PANI), sago starch, in situ polymerization, ultrasound irradiation, conductive composites

## Abstract

This work investigates the optimization of aniline content in polyaniline (PANI)/sago starch blends prepared via in situ oxidative polymerization under ultrasonic irradiation. Building upon our previous optimizations of pH and sonication time, this study focuses on the effect of aniline concentration (5–65 wt%) on electrical conductivity, morphological dispersion, and thermal stability. Various characterization techniques, including field emission scanning electron microscopy (FE-SEM), ultraviolet–visible (UV–Vis) spectroscopy, Fourier transform infrared (FT–IR) spectroscopy, and thermogravimetric analysis (TGA), confirm that a well-connected, conductive network forms at about 35 wt% aniline. Electrical conductivity measurements reveal a pronounced rise from ~1.6 × 10^−8^ to ~2.2 × 10^−3^ S/cm between 5 wt% and 35 wt% aniline. Conductivity stabilizes above this threshold due to PANI agglomeration. Morphological assessments confirm a shift from smooth, uniform blends at low aniline to rougher, void-filled surfaces when aniline exceeds 50 wt%. TGA shows improved thermal stability with increasing aniline content. These findings highlight an optimum aniline loading of ~35 wt% to achieve synergy between conductivity and structural integrity in biopolymer-based PANI/sago starch composites, offering a pathway to sustainable, high-performance biopolymer-based conductors for applications in sensors, flexible electronics, and electromagnetic shielding.

## 1. Introduction

Conducting polymers (CPs) have gained considerable interest over the past few decades due to their tunable conductivity and broad applicability in sensors, antistatic coatings, and electromagnetic shielding devices [1,2]. Among these conducting polymers, polyaniline (PANI) stands out for its ease of doping, relatively low-cost synthesis, and ability to achieve near-metallic conductivities under certain conditions [3,4]. However, doped PANI in its conducting form often suffers from brittleness and limited mechanical stability, which hampers its large-scale utilization [5,6]. To overcome these limitations, researchers have turned to blending PANI with other polymers—particularly those that are inexpensive and easy to process—to achieve biocompatible composites that retain good electrical performance [7,8].

Starch, including diverse types such as corn, potato, and sago, is an abundant natural biopolymer that has attracted interest as a matrix for conductive biopolymer blends due to its biodegradability and film-forming properties [9]. In particular, sago starch (*Metroxylon sagu*) is abundant in Southeast Asia but remains underutilized in high-value applications [10]. Unlike corn or potato starch, which are widely commercialized, sago presents unique challenges in processing and mechanical strength yet also offers a potentially lower cost and more regionally available resource [11]. Our earlier work demonstrated that PANI/sago starch blends could be synthesized using in situ oxidative polymerization under controlled conditions of pH and sonication time, resulting in composites with conductive and biodegradable characteristics. This work complements our previous studies [5,6], which explored the optimization of sonication time and pH to enhance the PANI dispersion and maintain the structural integrity of the sago starch matrix, and we show that aniline fraction is equally critical to achieving stable conductive networks and minimizing PANI agglomeration. In comparison to prior starch-based PANI composites (e.g., corn or potato starch) that often exhibit conductivities in the range of 10^−6^ to 10^−5^ S/cm [7,8,12], our sago-based blend can exceed 10^−3^ S/cm under optimal conditions, highlighting the cost-effectiveness and biodegradability of sago starch as a matrix. Moreover, PANI blends with conventional synthetic polymers (polystyrene, polyvinyl alcohol) typically display conductivities of 10^−6^ to 10^−3^ S/cm, so a well-tuned sago/PANI composite can be competitive with or superior to these existing materials in terms of electrical performance.

Recent advances in the field of PANI composites have focused on improving their mechanical strength, conductivity, and thermal stability by blending PANI with a variety of fillers, including carbon-based nanomaterials such as graphene [13,14,15], carbon nanotubes (CNTs) [16], and metal oxides (MnO_2_, ZnO) [17,18,19,20]. These fillers not only enhance the electrical properties of PANI but also improve mechanical robustness and thermal stability, making them more suitable for real-world applications such as supercapacitors, sensors, and flexible electronics. For instance, PANI–graphene oxide composites exhibit significant improvements in conductivity and electrochemical properties, driven by the cooperative effects between the filler and the polymer matrix. In these systems, the filler acts as a conduction bridge and improves the dispersion of PANI chains, reducing the tendency of PANI to form agglomerates that impede electron transport [8,20].

Additionally, functionalization and cross-linking of fillers play an important role in enhancing the mechanical properties and thermal stability of the composites. In particular, surface functionalization of graphene and CNTs, through chemical treatments, has been shown to significantly improve their dispersion in the polymer matrix and facilitate better interactions with PANI chains [20]. Cross-linkers such as glutaraldehyde have also been explored to enhance the structural integrity of PANI composites, promoting better network formation and improved mechanical properties [9].

Despite these promising developments, challenges remain in achieving a balance between conductivity and structural integrity in biodegradable matrices like sago starch. Insufficient aniline often leads to incomplete polymerization and suboptimal conductivity, whereas excessive aniline can result in agglomeration of PANI and reduced mechanical strength [7,8,11]. Excessive loading of PANI or fillers, while enhancing conductivity, can lead to agglomeration and a reduction in the mechanical stability of the composites, as seen in our earlier work and other studies [5,6]. Studies on PANI blends with synthetic polymers often report a percolation-like conductivity behavior as a function of PANI fraction, but relatively few studies detail the influence of higher aniline loading in biodegradable matrices, especially sago starch. Therefore, optimizing the loading ratio of PANI and fillers is crucial for maximizing conductivity while maintaining the biodegradability and mechanical strength of the final composite material [21]. Alongside compositional tuning, ultrasonic irradiation (UI) has become a valuable technique for polymerizing aniline in situ [22,23,24]. During sonication, cavitation bubbles introduce localized high-pressure, high-temperature microenvironments that enhance mass transfer and fragmentation of starch aggregates, yielding a more homogeneous distribution of growing PANI chains, lowering the percolation threshold, and potentially boosting mechanical properties. Combining UI with controlled monomer feed should therefore strengthen the interaction between starch and PANI. This balance between conductivity and structural properties becomes particularly important when considering the scalability and commercial viability of these materials for large-scale production. Moreover, the environmental stability of these composites under real-world conditions, including exposure to UV light, humidity, and temperature fluctuations, is a critical area of ongoing research [20].

Here, we present a detailed investigation on how varying aniline loadings (5–65 wt%) affect electrical conductivity, thermal stability, and film morphology in sago starch-based PANI blends under ultrasonic irradiation. The aim is to identify optimal aniline content that maximizes conductivity while preserving structural integrity, thereby contributing an additional layer of fine-tuning to our previously optimized synthesis conditions. We demonstrate that a sharp increase in conductivity occurs when aniline content reaches ~35 wt%, beyond which conductivity gains plateau and morphological defects (aggregates, voids) become significant. This work offers guidelines for designing green, high-performance polymer composites suitable for flexible electronics, sensors, and sustainable energy devices.

## 2. Materials and Methods

Aniline (monomer, Sigma-Aldrich, St. Louis, MO, USA), ammonium persulfate (Sigma-Aldrich, St. Louis, MO, USA), and hydrochloric acid (1 M HCl, Sigma-Aldrich, St. Louis, MO, USA) were used as received without additional purification. Sago starch (*Metroxylon sagu*) was obtained from a local supplier in Southeast Asia. Deionized (DI) water was used consistently across all experiments. The choice of HCl, APS, and sago starch followed the successful protocols in earlier work [5,6].

### 2.1. Preparation of PANI/Sago Blends

The preparation of PANI/sago starch blends followed a one-pot in situ oxidative polymerization method under ultrasonic irradiation. Initially, sago starch granules were soaked in deionized water overnight to facilitate hydration. The hydrated starch was then heated to approximately 90 °C for 15 min to achieve gelatinization. After cooling to 0–3 °C, the starch gel was placed in a probe sonicator bath set to deliver ultrasonic waves at a frequency of ~10 kHz. Varying amounts of aniline (5–65 wt% relative to the weight of sago starch) were introduced into the sonicator along with a fixed concentration of HCl (1 M) as the dopant acid. Ammonium persulfate (APS) solution was prepared freshly and added dropwise at a rate of 30 mL/h to initiate the oxidative polymerization of aniline. The polymerization was maintained for 2 h under sonication at a controlled pH of 6, adjusted as necessary by the controlled addition of NaOH. Upon completion of sonication, the dark-green suspension was allowed to rest undisturbed for 24 h to ensure complete polymerization. The final dispersion was cast onto clean glass plates and oven-dried at 60 °C for 48 h to form free-standing films. The dried films were carefully peeled off and rinsed with deionized water to remove any residual acid or unreacted monomers. To ensure reproducibility, each blend composition was prepared in triplicate, and the measured conductivity values (± standard deviation) were reproducible within 5%. For future standardization, controlling parameters such as monomer/oxidant feed rate, pH adjustment, and ultrasound energy input at larger scales will be crucial.

While this initial study focuses on optimizing PANI loading in a lab-scale ultrasonic polymerization setup, the approach is in principle translatable to larger batch processes. The reagents (aniline, HCl, APS, and sago starch) are readily available at industrial scales, and the mild reaction temperature is feasible using standard cooling loops. Provided that sonication or efficient mixing is maintained, an upscaled version could continuously produce PANI/sago blends in sheet or film forms. Additionally, sago starch is abundant and inexpensive in many Southeast Asian regions, suggesting an economically favorable path for larger-scale utilization.

Ultrasonication generates cavitation bubbles that collapse violently, creating localized microenvironments of elevated temperature and pressure. Under these conditions, oxidative polymerization is accelerated as radical initiation and chain propagation steps occur more rapidly [22,23,24]. Additionally, mechanical shear disrupts aggregated starch granules, improving monomer dispersion and helping PANI chains form more uniformly. This synergy reduces induction times, allows better control over molecular structure, and ultimately leads to higher conductivity in the resulting PANI/sago starch blends. Thus, ultrasonication not only serves as a physical mixing aid but also actively influences polymerization kinetics in our one-pot oxidative system. Table 1 shows the composition of PANI/sago blends with varying aniline content.

### 2.2. Characterization Techniques

All blend films were conditioned at ambient conditions (~25 °C, ~50% humidity) for at least 48 h prior to analysis. Field emission scanning electron microscopy (FE-SEM) was employed to examine the morphological characteristics of the blends. Samples were sputtered with a thin layer of gold to enhance conductivity and imaging quality. Energy-dispersive X-ray (EDX) mapping was performed to confirm the distribution of nitrogen, which indicates the presence of PANI within the sago starch matrix. Electrical conductivity was measured using a Jandel RM3000four-point probe system (Jandel, Leighton Buzzard, UK). Each conductivity value reported is an average of three independent measurements to ensure accuracy and reproducibility. Ultraviolet–visible (UV–Vis) spectroscopy was conducted using a PerkinElmer Lambda 1050 spectrophotometer (PerkinElmer, Waltham, MA, USA) over the range of 300–1000 nm. The presence of characteristic PANI absorption bands was monitored to assess the extent of doping and polymerization. Fourier transform infrared (FT-IR) spectroscopy was performed using a Nicolet 170SX spectrometer (Nicolet Instrument, Madison, WI, USA) in the range of 4000–400 cm^−1^. This analysis was conducted in attenuated total reflection (ATR) mode to investigate molecular interactions and structural changes within the blends. Thermogravimetric analysis (TGA) was carried out using a PerkinElmer Pyris-7 TGA system (PerkinElmer, Waltham, MA, USA). Samples were heated from 40 to 900 °C at a rate of 20 °C/min under a nitrogen atmosphere (20 mL/min). The analysis provided insights into the decomposition stages and residual char content of the blends. Although no formal rheological measurements were taken, we anticipate that rheology will further clarify how sago starch’s gelatinization and ultrasonication affect polymerization kinetics. Future work will incorporate rheological evaluations to better link sago starch sol-gel transitions with polymerization kinetics.

## 3. Results and Discussion

### 3.1. Morphological Evolution with Aniline Content

**Figure 1:**
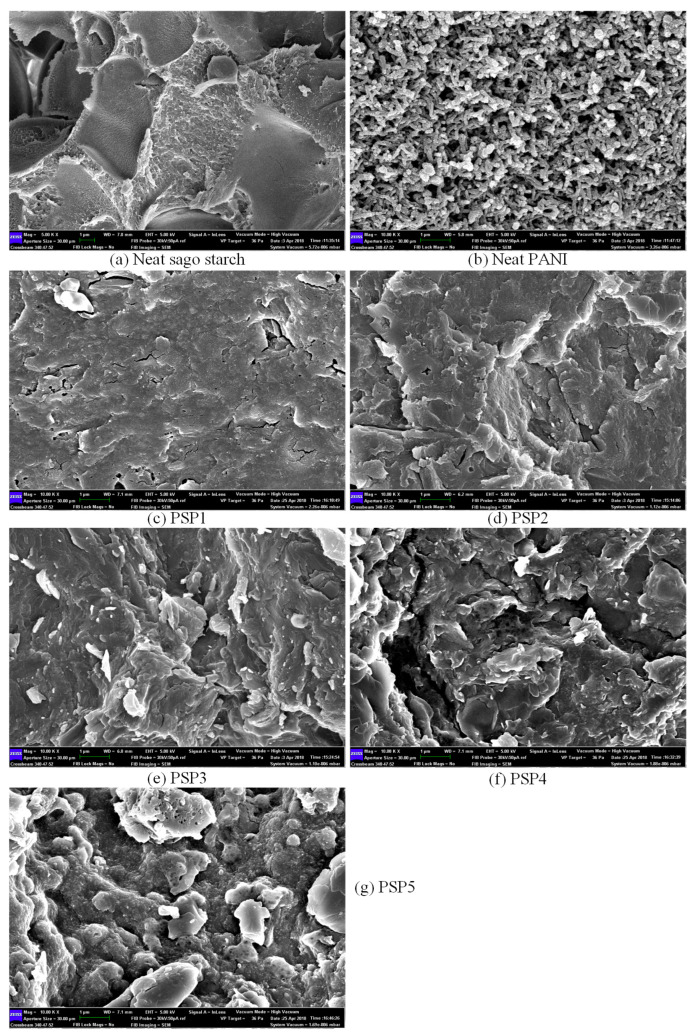
FE-SEM micrograph of (**a**) neat sago starch (**b**) neat PANI (**c**) PSP1 (**d**) PSP2 (**e**) PSP3 (**f**) PSP4 (**g**) PSP5.

Figure 1 shows the FE-SEM micrographs of neat sago starch, neat PANI, and PANI/sago starch blends (PSP1 to PSP5), revealing the progressive impact of aniline content on surface morphology. The images illustrate that neat sago starch exhibits a smooth and well-defined surface, while neat PANI displays cylindrical rod-like structures, consistent with typical PANI morphology [25]. As seen in blends PSP1 (5 wt%) and PSP2 (20 wt%), the introduction of PANI results in a rougher surface compared to neat sago, indicating successful polymerization. These lower aniline fractions maintain relatively uniform and smooth morphologies, suggesting effective dispersion of PANI within the sago matrix.

At 35 wt% aniline content (PSP3), the morphology becomes more interconnected, indicative of a well-formed conductive network. This blend shows a uniform distribution of PANI chains without significant agglomeration. The formation of such an interconnected network is crucial for effective charge transport, as it facilitates continuous pathways for electron movement [26]. However, at higher aniline loadings (50 wt% and 65 wt%, PSP4 and PSP5), the FE-SEM images reveal the formation of voids and lumps. This agglomeration is due to the inability of sago starch to encapsulate the excessive PANI, leading to overgrowth and clustering of the polymer chains. The presence of voids and lumps can disrupt the uniform conductive network, potentially creating barriers to electron flow and reducing overall conductivity [27].

**Figure 2:**
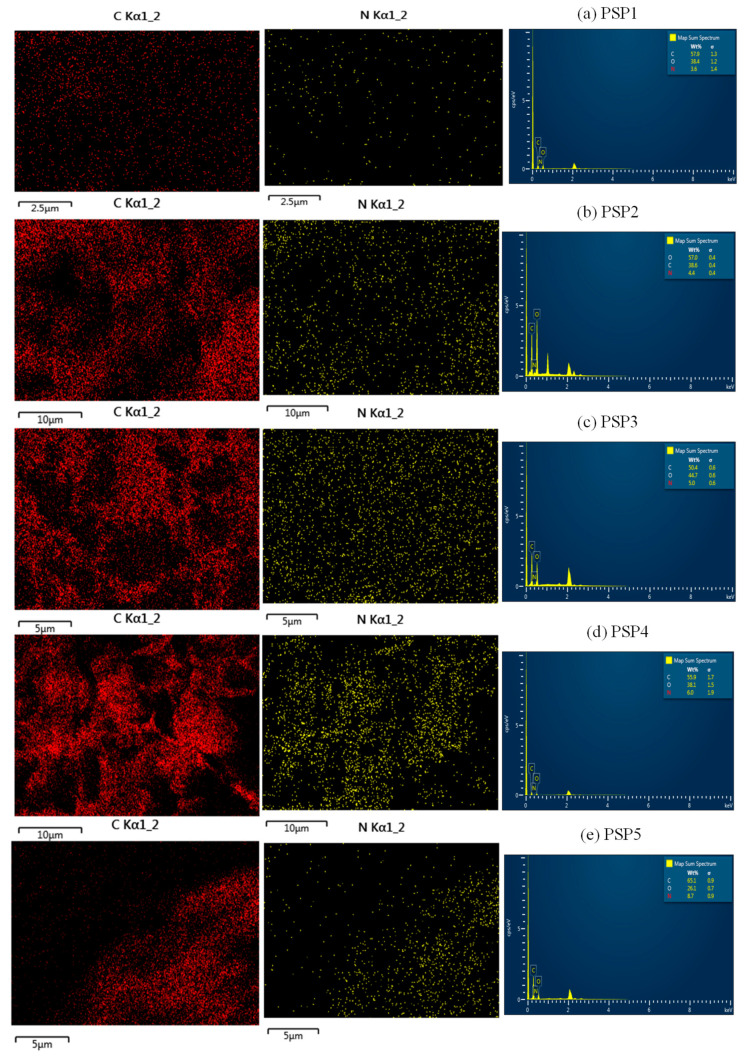
Illustration of the EDX mapping area of the PANI/sago blends of different aniline contents: (**a**) PSP1, (**b**) PSP2, (**c**) PSP3, (**d**) PSP4, (**e**) PSP5.

Figure 2 further confirms these observations through energy-dispersive X-ray (EDX) mapping, which illustrates a uniform distribution of nitrogen atoms in PSP1 to PSP4, indicating consistent PANI incorporation. In PSP5, the nitrogen distribution becomes non-uniform, reflecting the agglomeration observed in the FE-SEM micrographs. The increase in the atomic percentage of nitrogen with higher aniline content, as shown in Table 1, validates the progressive incorporation of PANI into the sago matrix. However, in PSP5, the non-uniform nitrogen distribution correlates with the morphological agglomeration, suggesting that excessive PANI leads to phase separation and clustering [28].

### 3.2. Electrical Conductivity and Percolation

**Figure 3:**
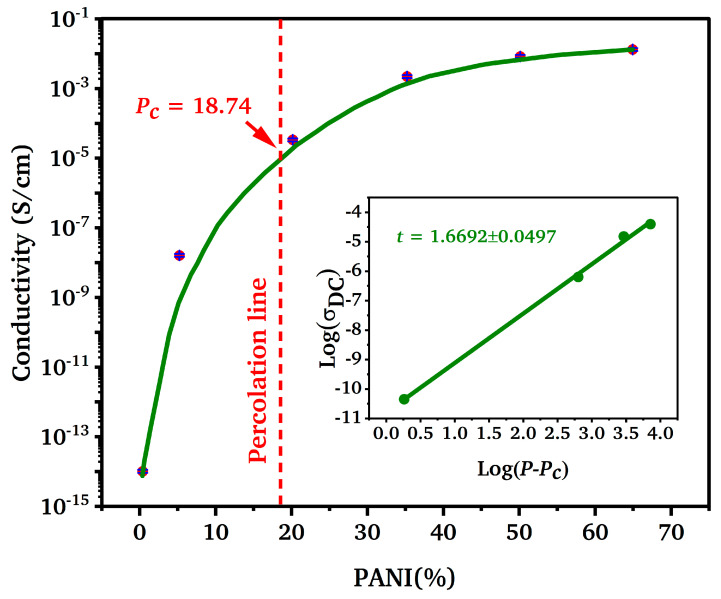
Effect of aniline content on the electrical conductivity of PANI/sago blends.

Figure 3 illustrates the electrical conductivity of PANI/sago blend films as a function of aniline content. A notable percolation-like behavior is observed, with conductivity increasing sharply from PSP1 (5 wt%, 1.59 × 10^−8^ S/cm) to PSP3 (35 wt%, 2.17 × 10^−3^ S/cm), followed by a plateau and slight decline in PSP4 (50 wt%, 8.28 × 10^−3^ S/cm) and PSP5 (65 wt%, 1.26 × 10^−2^ S/cm).

This trend can be attributed to the formation of a connected conductive network at the critical percolation threshold (~18.74 wt%), as evidenced by the scaling law fitting (*σ* = *c*(*P* − *P_c_*)*^t^*, *R*^2^ = 0.99, *t* = 1.6) [29]. The percolation threshold was calculated from fitting the experimental data using a plot of log *σ* vs. log (*P*−*P_C_*). The fit had a very good correlation (*R*^2^ = 0.99), and the value of the exponent t is 1.6, which represents three-dimensional conductivity. The sharp increase up to 35 wt% indicates that ultrasonic irradiation effectively facilitates the dispersion and polymerization of aniline, enhancing charge carrier mobility and interparticle connectivity. The percolation exponent (*t* = 1.6) aligns closely with theoretical predictions for three-dimensional conductive networks, suggesting that the PANI chains form a robust, interconnected framework within the sago starch matrix [7].

However, beyond 35 wt% aniline, the conductivity does not continue to increase proportionally. Instead, it stabilizes and even shows a slight decline at higher aniline contents. This behavior is indicative of PANI agglomeration, as observed in the FE-SEM micrographs (Figure 1). The excessive PANI content overwhelms the encapsulating capacity of sago starch, leading to the formation of PANI clusters that disrupt the uniform conductive pathways. This agglomeration not only impedes further charge transport but may also introduce scattering centers that reduce overall conductivity. Compared to previous studies [30], which reported similar percolation thresholds in PANI/polymer blends, our optimized aniline content demonstrates a lower percolation threshold (~18.74 wt%) attributed to the enhanced dispersion achieved through ultrasonic irradiation. This low threshold signifies efficient interaction between PANI and sago starch, promoting a robust conductive network with minimal monomer excess.

The high conductivity achieved at PSP3 (~2 × 10^−3^ S/cm) surpasses typical values reported for similar systems (10^−6^ to 10^−7^ S/cm) by Bhadra et al. [7], underscoring the efficacy of ultrasonic irradiation in promoting superior conductive networks. This superior conductivity is not only beneficial for applications requiring high electrical performance but also reflects the successful integration of PANI within a sustainable, biopolymer matrix. This synergy of pH, sonication, and aniline fraction thus provides a multifactor approach for tailoring mechanical integrity, conductivity, and film uniformity, enabling potential applications in electromagnetic shielding, flexible sensors, and low-cost conductive films.

### 3.3. Ultraviolet–Visible (UV–Vis) Spectroscopy

**Figure 4:**
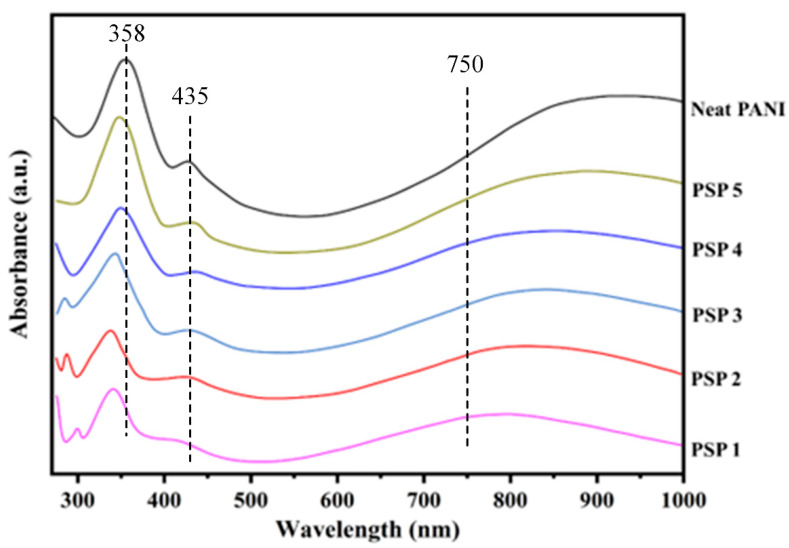
UV–Vis spectra of PANI, sago, and PANI/sago blends with varying aniline content.

Figure 4 presents the UV–Vis spectra of PANI, sago starch, and PANI/sago starch blends with varying aniline content. Ultraviolet–visible (UV–Vis) spectroscopy was employed to monitor the electronic transitions associated with PANI in the sago starch matrix. All blends exhibit three characteristic absorption bands of PANI: a π–π* transition near ~358 nm, an exciton-coupled π-polaron transition around ~435 nm, and a polaron–π* transition extending beyond ~750 nm [31].

At lower aniline loadings (PSP1 and PSP2), the absorption bands are less intense, indicating limited PANI formation. As the aniline content increases to 35 wt% (PSP3), the polaronic bands become significantly more pronounced, confirming the establishment of an extensive conductive network. The slight flattening of the polaron–π* transition in PSP5 may result from over-oxidation or polymer chain entanglement, affecting the uniform distribution of PANI within the matrix.

The intensity of the polaron transitions increases systematically with aniline fraction, reflecting the greater presence of doped PANI chains within the blend. The shifting of the absorption bands towards the UV region and the increase in their intensity suggest the dominance of PANI in the blend, enhancing the electronic conduction properties [32]. These spectroscopic findings are in strong agreement with the electrical conductivity results, where optimal PANI network formation corresponds to maximum conductivity. The enhanced absorption in the polaron–π* transition region indicates increased charge delocalization within the PANI chains, contributing to higher conductivity.

### 3.4. Fourier Transform Infrared (FT-IR) Spectroscopy

**Figure 5:**
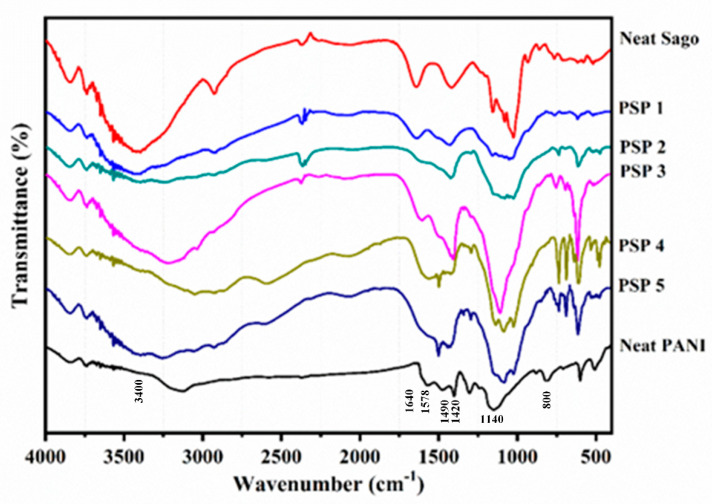
FT-IR spectrums of PANI, sago, and PANI/sago blends with varying aniline content.

Figure 5 presents the FT-IR spectra of neat PANI, neat sago starch, and the PANI/sago blends at varying aniline contents (5–65 wt%). The characteristic PANI signals—quinoid ring stretching (~1578 cm^−1^), benzenoid ring stretching (~1490 cm^−1^), and the prominent N⁺–H stretching near 1140 cm^−1^—remain relatively weak or partially obscured in PSP1 (5 wt%) and PSP2 (20 wt%), reflecting the limited incorporation of PANI at low monomer loadings [33]. Moreover, the pronounced starch peaks in these blends, including the strong C–O stretch bands around 1000–1200 cm^−1^ and the broad O–H stretch near 3400 cm^−1^, indicate that sago starch dominates the matrix at lower aniline levels [5,6].

A significant shift in spectral features emerges once the aniline content exceeds ~35 wt% (PSP3), where the PANI peaks become more defined and start to merge with or slightly shift the starch bands. Specifically, quinoid- and benzenoid-related absorptions intensify around 1578–1490 cm^−1^, demonstrating enhanced conjugation and effective polymerization within the sago starch network [5,33]. Similarly, the overlap and broadening of the starch-related bands at ~1640 cm^−1^ (commonly associated with water absorption) and ~1420 cm^−1^ suggest increasing PANI–starch interaction, possibly through hydrogen bonding between the amine sites of PANI and the hydroxyl groups of starch. At even higher aniline contents (PSP4 and PSP5), the spectra reveal more dominant PANI signals—particularly near 800 cm^−1^ (para-substituted ring modes)—accompanied by partial suppression or shifting of starch peaks, consistent with the morphological finding of PANI-rich domains [7]. This spectral evolution confirms that optimal conductivity in these blends correlates with a balanced molecular interplay between starch and PANI chains, and that excessive aniline leads to overgrowth of PANI, as evidenced by broadened peaks and subsequent agglomeration noted in the SEM results.

### 3.5. Thermogravimetric Analysis (TGA)

**Figure 6:**
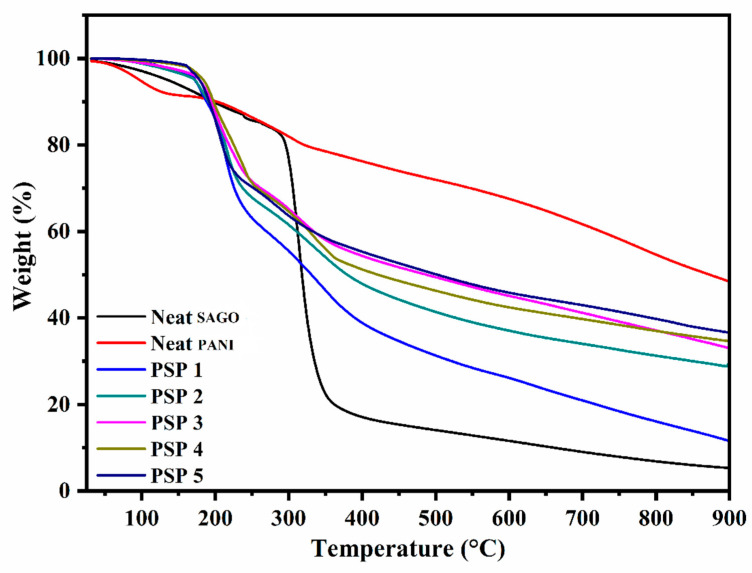
TG analysis of PANI, sago, and PANI/sago blends of varying aniline content.

Figure 6 compares the thermogravimetric (TG) profiles of neat sago starch, neat PANI, and the PANI/sago starch blends with varying aniline contents under a nitrogen atmosphere. Neat sago starch exhibits a sharp drop between 290 °C and 390 °C (from ~84 wt% to ~18 wt%), consistent with the onset of starch backbone degradation [34]. In contrast, neat PANI shows a multi-step mass loss, beginning with moisture and volatile impurities below ~180 °C, followed by dopant acid/oligomer loss in the range of 180–240 °C, then further degradation of the PANI–dopant complex between 240 °C and 380 °C, and finally exothermic decomposition of the polymer backbone above ~380 °C.

All PANI/sago blends exhibit an initial weight loss (40–180 °C) attributed to residual moisture, free acids, and unreacted monomers generated during the oxidative polymerization [35]. A second weight-loss step (~180–240 °C) reflects the partial degradation of short-chain oligomers, which increases slightly with aniline content. The third and fourth degradation steps (beyond ~240 °C) shift to progressively higher temperatures in blends containing more aniline, suggesting enhanced thermal stability through stronger PANI–HCl interactions and partial cross-linking effects at elevated temperatures [36]. Moreover, the final char residue grows with higher aniline loadings, as greater PANI content fosters increased carbonization under inert conditions. Taken together, the TG results demonstrate that introducing PANI into sago starch significantly improves thermal stability; at an optimal aniline content (~35 wt% or above), the blends display a higher onset of major decomposition and a larger residual mass compared to neat sago starch, confirming the protective role of PANI in the starch matrix.

## 4. Conclusions

This study successfully identifies the optimal aniline content for enhancing the electrical conductivity and thermal stability of PANI/sago starch blends under fixed conditions of pH 6 and sonication time of 2 h. The electrical conductivity of the blends increases sharply up to 35 wt% aniline, reaching approximately ~2.17 × 10^−3^ S/cm, and stabilizes thereafter due to PANI agglomeration. Morphological assessments via FE-SEM confirm the formation of a well-connected PANI network at optimal aniline loading, while higher concentrations lead to lumps and void formation. UV–Vis and FT-IR spectroscopy confirm the increased presence and interaction of PANI within the sago starch matrix, and TGA demonstrates improved thermal stability with higher aniline content. Identifying 35 wt% aniline as the optimal loading provides a balanced approach to achieving high conductivity and structural integrity in PANI/sago starch blends. This finding, combined with earlier optimizations of pH and sonication conditions, offers a comprehensive framework for the scalable production of strong, conducting PANI/sago composites. Future research will incorporate carbon-based fillers (e.g., CNTs or graphene) and cross-linkers to further improve mechanical performance while retaining the biopolymer’s film-forming properties. Preliminary tests suggest such hybrid systems may also preserve conductivity. In addition, we plan to investigate the UV/weathering stability and long-term biodegradation behavior of these films in real-world conditions, which will broaden their potential for advanced applications in flexible electronics, biosensors, and EMI shielding.

## Figures and Tables

**Table 1:** Compositions of PANI/sago blends with varying aniline content.

Sample	Aniline Content (wt%)	Atomic Percentage of Nitrogen (wt%)	Electrical Conductivity (S/cm)
Neat PANI	-	-	1.78 × 10^−2^
Neat Sago	-	-	1 × 10^−14^
PSP1	5	3.6	1.5923 × 10^−8^
PSP2	20	4.4	3.3554 × 10^−5^
PSP3	35	5.0	0.00217
PSP4	50	6.0	0.00828
PSP5	65	8.7	0.01261

## Data Availability

The data that support the findings of this study are available from the corresponding author upon reasonable request.

## References

[1] MacDiarmid A.G. (2001). Synthetic metals: A novel role for organic polymers (Nobel lecture). Angew. Chem. Int. Ed..

[2] Inzelt G. (2012). Conducting Polymers: A New Era in Electrochemistry.

[3] Gulrez S.K.H., Al-Assaf S., Phillips G.O., Al-Lohedan H.A., Ghezza L. (2014). A review on electrically conductive polypropylene and polyethylene. Polym. Compos..

[4] Saikia J.P., Ganguly M., Majumdar G., Dutta P.K. (2010). Biocompatible novel starch/polyaniline composites: Characterization, anti-cytotoxicity and antioxidant activity. Colloids Surf. B Biointerfaces.

[5] Ali Mohsin M.E., Shrivastava N.K., Arsad A., Basar N., Hassan A. (2019). The effect of sonication time on the properties of electrically conductive PANI/sago starch blend prepared by the one-pot synthesis method. Front. Mater..

[6] Ali Mohsin M.E., Shrivastava N.K., Arsad A., Basar N., Hassan A. (2020). The effect of pH on the preparation of electrically conductive and physically stable PANI/sago blend film via in situ polymerization. Front. Mater..

[7] Bhadra J., Al-Thani N.J., Madi N.K., Al-Maadeed M.A. (2017). Effects of aniline concentrations on the electrical and mechanical properties of polyaniline-polyvinyl alcohol blends. Arab. J. Chem..

[8] Prabhakar R., Kumar D. (2019). Studies on polyacrylate-starch/polyaniline conducting hydrogel. Curr. Smart Mater..

[9] Uthumporn U., Wahidah N., Karim A. (2014). Physicochemical properties of starch from sago (*Metroxylon sagu*) palm grown in mineral soil at different growth stages. IOP Conference Series: Materials Science and Engineering.

[10] Lu D., Xiao C., Xu S. (2009). Starch-based completely biodegradable polymer materials. Express Polym. Lett..

[11] Razak S.I.A., Anuar F.H., Rahman W.A.W.A., Awang M., Bakar A.A.A. (2013). Polyaniline-coated kenaf core and its effect on the mechanical and electrical properties of epoxy resin. Compos. Interfaces.

[12] Janaki V., Vijayaraghavan K., Oh B.-T., Lee K.-J., Muthuchelian K., Ramasamy A.K. (2012). Starch/polyaniline nanocomposite for enhanced removal of reactive dyes from synthetic effluent. Carbohydr. Polym..

[13] Li X.P., Ma G., Nie Y., Song Y., Li H., Niu Q.J., Qiu H., Hao X., Liu S. (2022). Reshapable MXene/graphene oxide/polyaniline plastic hybrids with patternable surfaces for highly efficient solar-driven water purification. Adv. Funct. Mater..

[14] Zhang Y., Zhou C.-G., Yan X., Gao H.-L., Gao K.-Z., Cao Y. (2023). Synthesis of Nafion-reduced graphene oxide/polyaniline as novel positive electrode additives for high-performance lead-acid batteries. Electrochim. Acta.

[15] Xie Z., Wang J., Yeow J.T.W. (2022). Doped polyaniline/graphene composites for photothermoelectric detectors. ACS Appl. Nano Mater..

[16] Singh G., Kumar Y., Husain S. (2023). Fabrication of high energy density symmetric polyaniline/functionalized multiwalled carbon nanotubes supercapacitor device with swift charge transport in different electrolytic mediums. J. Energy Storage.

[17] Lal Meena P., Kumar Saini J. (2023). Synthesis of polymer–metal oxide (PANI/ZnO/MnO₂) ternary nanocomposite for effective removal of water pollutants. Results Chem..

[18] Nguyet T.T., Van Duy L., Nguyet Q.T.M., Bui D.Q., Duong V.T., Bach L.G., Vo D.-V.N., Trinh Q.T., Nguyen T.H. (2023). Novel synthesis of PANI/ZnO nanohybrid for enhanced NO₂ gas sensing performance at low temperatures. J. Electron. Mater..

[19] Wang L., Guo C., Lv H., Zhang H., Song R., Lu Z., Hu X. (2023). Construction of polyaniline/MnO₂ core–shell nanocomposites in carbonized wood tracheids for high-performance all-solid-state asymmetric supercapacitors. Appl. Surf. Sci..

[20] Beygisangchin M., Hossein Baghdadi A., Kartom Kamarudin S., Abdul Rashid S., Jakmunee J., Shaari N. (2024). Recent progress in polyaniline and its composites: Synthesis, properties, and applications. Eur. Polym. J..

[21] Nan C.-W., Shen Y., Ma J. (2010). Physical properties of composites near percolation. Annu. Rev. Mater. Res..

[22] Xia H., Wang Q. (2002). Ultrasonic irradiation: A novel approach to prepare conductive polyaniline/nanocrystalline titanium oxide composites. Chem. Mater..

[23] Jing X., Liu C., Fu S. (2006). Polyaniline nanofibers prepared with ultrasonic irradiation. J. Polym. Sci. A Polym. Chem..

[24] Shabana S., Jan K., Hussain S.Z., Rather S.A., Majeed A., Ali U., Asiri A.M., Manzoor N., Bhat T.A. (2019). Ultrasound-assisted acid hydrolyzed structure modification and loading of antioxidants on potato starch nanoparticles. Ultrason. Sonochem..

[25] Tiwari I., Singh K. (2012). In situ synthesis of polymer nanocomposites from PANI/PAA/MWCNTs: Analysis and characterization. Int. J. Polym. Anal. Charact..

[26] Chen F., Liu P. (2011). High electrically conductive polyaniline/partially phosphorylated poly(vinyl alcohol) composite films via aqueous dispersions. Macromol. Res..

[27] Wang H., Cheng Z., Li D., Ren Y., Yu J., Liu Q., Zhang J., Shen D. (2017). Facile approach to fabricate waterborne polyaniline nanocomposites with environmental benignity and high physical properties. Sci. Rep..

[28] Mumtaz M., Li P., Jenkins M.J., Rimmer S. (2009). Synthesis of polyaniline nano-objects using poly(vinyl alcohol)-, poly(ethylene oxide)-, and poly[(N-vinyl pyrrolidone)-co-(vinyl alcohol)]-based reactive stabilizers. Langmuir.

[29] Stauffer D., Aharony A. (2018). Introduction to Percolation Theory.

[30] Shrivastava N.K., Wahit M.U., Ismail H., Zainuddin N., Handan T.E., Rosli M.F., Othman N. (2014). An approach to reduce the percolation threshold of MWCNT in ABS/MWCNT nanocomposites through selective distribution of CNT in ABS matrix. RSC Adv..

[31] Borah R., Banerjee S., Kumar A. (2014). Surface functionalization effects on structural, conformational, and optical properties of polyaniline nanofibers. Synth. Met..

[32] Bhadra J., Madi N.K., Al-Thani N.J., Al-Maadeed M.A. (2019). Fabrication of polyaniline–graphene/polystyrene nanocomposites for flexible gas sensors. RSC Adv..

[33] Ping Z. (1996). In situ FTIR–attenuated total reflection spectroscopic investigations on the base–acid transitions of polyaniline. Base–acid transition in the emeraldine form of polyaniline. J. Chem. Soc. Faraday Trans..

[34] Neelgund G.M., Oki A. (2011). A facile method for the synthesis of polyaniline nanospheres and the effect of doping on their electrical conductivity. Polym. Int..

[35] Laska J., Zak K., Proń A. (1997). Conducting blends of polyaniline with conventional polymers. Synth. Met..

[36] Kabomo T.M., Scurrell M.S. (2016). The effect of protonation and oxidation state of polyaniline on the stability of gold nanoparticles. Eur. Polym. J..

